# Role of Mouse Organic Cation Transporter 2 for Nephro- and Peripheral Neurotoxicity Induced by Chemotherapeutic Treatment with Cisplatin

**DOI:** 10.3390/ijms241411486

**Published:** 2023-07-14

**Authors:** Anna Hucke, Rita Schröter, Cecilia Ceresa, Alessia Chiorazzi, Annalisa Canta, Sara Semperboni, Paola Marmiroli, Guido Cavaletti, Burkhard Gess, Giuliano Ciarimboli

**Affiliations:** 1 Experimentelle Nephrologie, Medizinische Klinik D, Universitätsklinikum Münster, 48149 Münster, Germany; a_huck01@uni-muenster.de (A.H.); ritas@uni-muenster.de (R.S.); 2 Experimental Neurology Unit, Department of Medicine and Surgery, University of Milano Bicocca, 20900 Monza, Italy; cecilia.ceresa@gmail.com (C.C.); alessia.chiorazzi@unimib.it (A.C.); annalisa.canta@unimib.it (A.C.); sarasemperboni@gmail.com (S.S.); paola.marmiroli@unimib.it (P.M.); guido.cavaletti@unimib.it (G.C.); 3 Fondazione IRCCS San Gerardo dei Tintori, 20900 Monza, Italy; 4 Department of Neurology, University Hospital Münster, 48149 Münster, Germany; burkhard.gess@evkb.de; 5 Department of Neurology, Evangelisches Klinikum Bethel, University of Bielefeld, 33617 Bielefeld, Germany

**Keywords:** transporters, cisplatin, toxicity, kidneys, dorsal root ganglia, peripheral neurotoxicity

## Abstract

Cisplatin (CDDP) is an efficient chemotherapeutic agent broadly used to treat solid cancers. Chemotherapy with CDDP can cause significant unwanted side effects such as renal toxicity and peripheral neurotoxicity. CDDP is a substrate of organic cation transporters (OCT), transporters that are highly expressed in renal tissue. Therefore, CDDP uptake by OCT may play a role in causing unwanted toxicities of CDDP anticancer treatment. In this study, the contribution of the mouse OCT2 (mOCT2) to CDDP nephro- and peripheral neurotoxicity was investigated by comparing the effects of cyclic treatment with low doses of CDDP on renal and neurological functions in wild-type (WT) mice and mice with genetic deletion of OCT2 (OCT2^−/−^ mice). This CDDP treatment protocol caused significant impairment of kidneys and peripherical neurological functions in WT mice. These effects were significantly reduced in OCT2^−/−^ mice, however, less profoundly than what was previously measured in mice with genetic deletion of both OCT1 and 2 (OCT1-2^−/−^ mice). Comparing the apparent affinities (IC_50_) of mOCT1 and mOCT2 for CDDP, the mOCT1 displayed a higher affinity for CDDP than the mOCT2 (IC_50_: 9 and 558 µM, respectively). Also, cellular toxicity induced by incubation with 100 µM CDDP was more pronounced in cells stably expressing mOCT1 than in cells expressing mOCT2. Therefore, in mice, CDDP uptake by both OCT1 and 2 contributes to the development of CDDP undesired side effects. OCT seem to be suitable targets for establishing treatment protocols aimed at decreasing unwanted CDDP toxicity and improving anticancer treatment with CDDP.

## 1. Introduction

Cisplatin (cis-diamminedichloroplatinum-CDDP) is a widely used chemotherapeutic agent that is an important part of modern oncology [1]. Although CDDP is potent against solid cancer, it can also damage non-target cells and cause significant unwanted toxicities that can compromise the success of antineoplastic therapy and affect the quality of life of patients after cancer treatment. The most common adverse effects of CDDP cancer therapy are nephrotoxicity, ototoxicity, and peripheral neurotoxicity [2]. CDDP-associated nephrotoxicity is a dose-limiting effect, requiring dose adjustment or even discontinuation of chemotherapy. Acute kidney injury (AKI) has been observed in ca 70% of pediatric [3] and 30% of adult [4] patients treated with CDDP. Nearly 80% of children treated with CDDP develop ototoxicity [5], for which there is no cure. Of course, this toxicity hampers children’s psychological and social development. Peripheral neurotoxicity develops in up to 30–50% of patients treated with CDDP, independent of the doses and treatment protocol [6]. This off-target CDDP effect is due to its accumulation in the dorsal root ganglia (DRG) [7], where it induces apoptosis and leads to atrophy or loss of peripheral sensory neurons. Nevertheless, cancer chemotherapy with CDDP is still widely used because it is very effective in the treatment of solid tumors, especially testicular cancer, where CDDP treatment combined with surgery is considered curative [8].

To date, there is no specific treatment to prevent unwanted CDDP toxicities. Therefore, elucidation of the mechanisms contributing to CDDP toxicities is important in order to develop appropriate protective strategies. 

The main mechanism of anticancer action of CDDP is the formation of intra- and inter-strand adducts with nuclear and mitochondrial DNA [9], which activate cellular DNA-damage responses and increase oxidative stress, ultimately leading to cell apoptosis [10]. However, CDDP cellular damage can also result from a direct interaction of CDDP with proteins, as has been shown, for example, for the CDDP adduct of the Iron Regulatory Protein 2 [11]. It is clear that CDDP exerts its toxic effects intracellularly and, therefore, must first cross the plasma membrane to reach its targets. As CDDP is a hydrophilic drug [12], its movement across the plasma membrane may be facilitated by transporters. Indeed, several transport proteins have been identified that accept CDDP as a substrate and can mediate its uptake or efflux into or out of the cell [13]. A particular role in the development of undesired CDDP toxicities has been attributed to the organic cation transporter 2, as it is a concentrative transporter that is highly expressed in tissues such as the proximal tubules of the kidney [14], the cochlear hair, and stria vascularis cells [15] and also in DRGs [16]. In this work, the role of mouse organic cation transporter 2 (mOCT2) in CDDP nephrotoxicity and peripheral neurotoxicity was investigated by comparing changes in renal and neurological functions in wild-type (WT) mice and mice with global genetic deletion of OCT2 under chronic cyclic CDDP treatment, a treatment protocol similar to cancer chemotherapeutic protocols used in patients. 

## 2. Results

**Figure 1:**
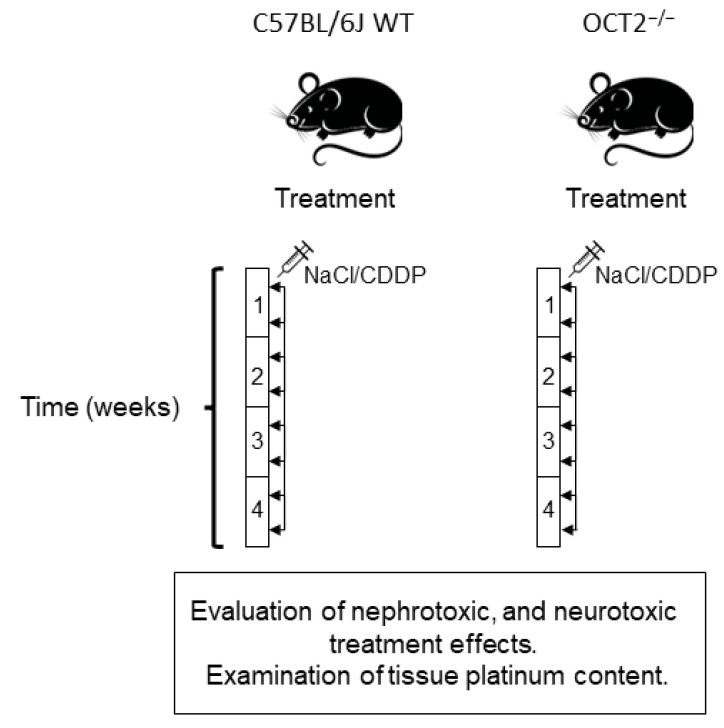
The treatment protocol for the mice is depicted in this figure. Male C57BL/6J mice (wild-type, WT) and male OCT2^−/−^ mice in the C57BL/6J background were subjected to intraperitoneal (i.p.) injections twice a week for a duration of 4 weeks. The injections consisted of either 4 mg/kg of cisplatin (CDDP) or an equivalent volume of vehicle (NaCl) as a control. Upon completion of the 4-week treatment period, measurements were taken to evaluate kidney and neurological function. Subsequently, the animals were sacrificed, and their organs were collected for further analysis.

The study involved two groups of mice, OCT2^−/−^ mice on the C57BL/6J background (referred to as OCT2^−/−^ mice) and C57BL/6J-wild-type mice (referred to as WT mice). Both groups were subjected to a treatment regimen consisting of twice-weekly intraperitoneal (i.p.) injections of 4 mg/kg body weight (BW) of CDDP, resulting in a cumulative dose of 32 mg/kg BW. It is important to note that this dosage was lower compared to what was used in acute toxicity experiments [15,17] but similar to the treatment protocol followed by patients, which involves repeated cycles of the highest possible dose [17]. Control experiments were conducted using repeated i.p. injections of a vehicle solution, specifically normal saline (NaCl). At the conclusion of the treatment, renal and neurological functions were assessed prior to harvesting the organs affected by CDDP toxicity (refer to Figure 1).

**Figure 2:**
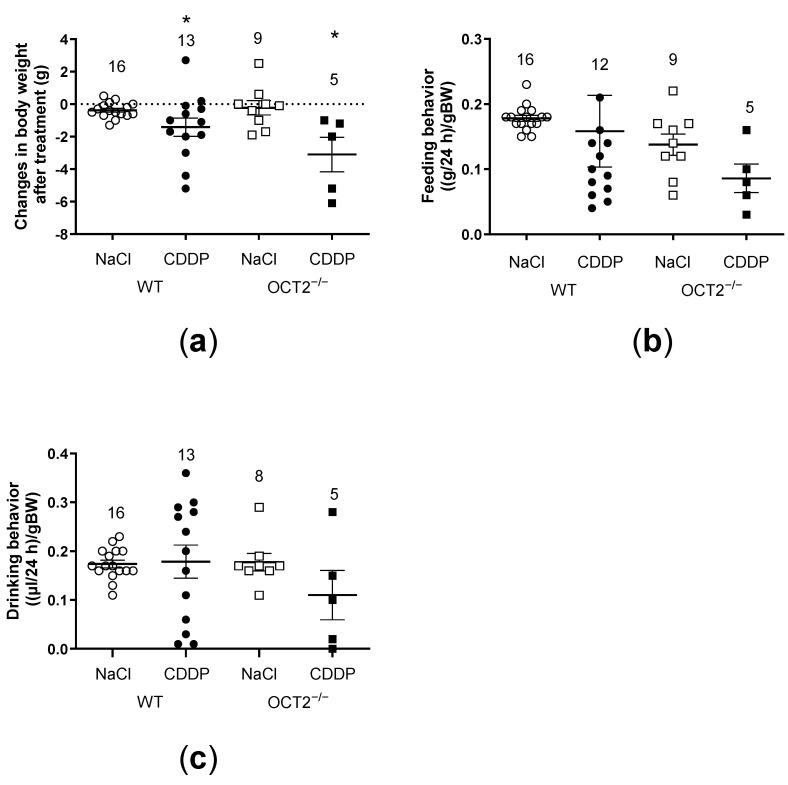
In this figure, changes in various parameters are depicted for both WT and OCT2^−/−^ mice, who were subjected to different treatments involving either vehicle (normal saline, NaCl) or CDDP. Each symbol in the figure represents an individual animal, while the horizontal bars indicate the mean value ± SEM of the observations. The effects of CDDP treatment on body weight (BW) are shown in panel (**a**), feeding behavior in panel (**b**), and drinking behavior in panel (**c**). The results demonstrate that CDDP treatment led to a significant decrease in body weight for both WT and OCT2^−/−^ mice, compared to the animals treated with the vehicle (NaCl) (panel (**a**)), * *p* = 0.0098, determined by the two-way ANOVA test). However, no significant differences were observed in feeding behavior (panel (**b**)) and drinking behavior (panel (**c**)) among the various treatment groups.

**Figure 3:**
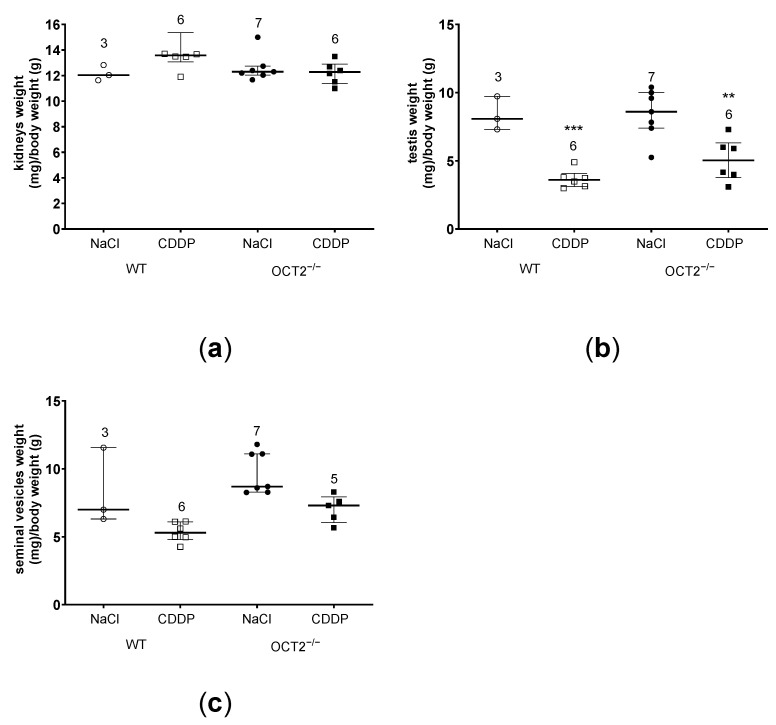
In this figure, the changes in the weights of kidneys (panel (**a**)), testis (panel (**b**)), and seminal vesicles (panel (**c**)) are presented, resulting from treatment with either vehicle (NaCl) or CDDP in both WT (represented by open symbols) and OCT2^−/−^ mice (represented by closed symbols). Each symbol in the figure corresponds to an individual animal, while the horizontal bars represent the median value along with the interquartile range of the observations. The results demonstrate that CDDP treatment significantly decreased the weight of the testis in both WT and OCT2^−/−^ mice when compared to the testis weight of vehicle-treated animals (panel (**b**), *** *p* = 0.0006 for WT mice and ** *p* = 0.0067 for OCT2^−/−^ mice, determined by the two-way ANOVA test). The numbers displayed above the experimental groups indicate the number of animals used in each group.

CDDP treatment caused a statistically significant decrease in body weight in both OCT2^−/−^ and WT mice (Figure 2a) but no significant change in feeding and drinking behavior (Figure 2b,c). Interestingly, compared to vehicle administration, CDDP chronic treatment did not affect kidney weight but significantly decreased testis weight in both OCT2^−/−^ and WT mice (Figure 3a,b). The weights of seminal vesicles showed a trend to decrease after CDDP treatment, but this effect did not reach statistical significance (Figure 3c). Taken together, these results suggest that OCT2 may not be involved in the testicular action of CDDP.

**Figure 4:**
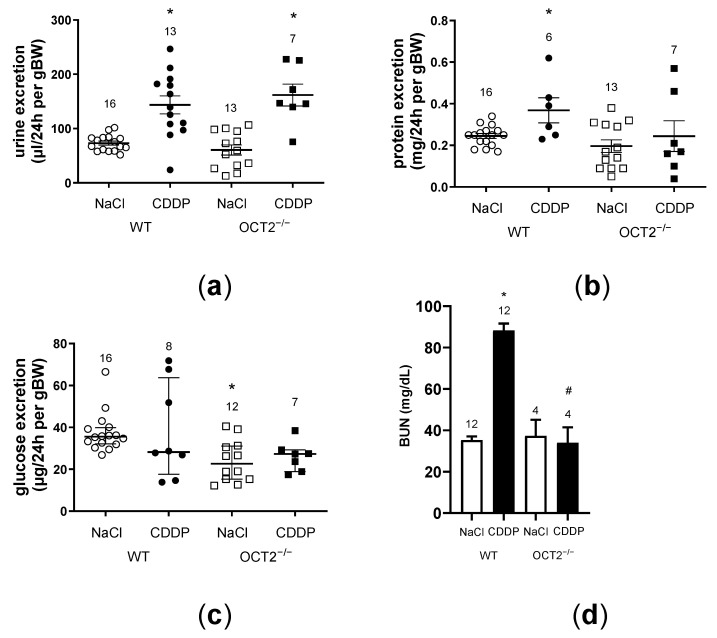
In this figure, the changes in urine excretion, protein excretion, glucose excretion, and blood urea nitrogen (BUN) levels are depicted as a result of treatment with CDDP or vehicle (NaCl) in both WT and OCT2^−/−^ mice. Each symbol in the figure represents an individual animal, and the numbers above the columns indicate the number of animals in each group. Panel (**a**): The urine excretion significantly increased in both WT and OCT2^−/−^ mice treated with CDDP compared to the control animals (NaCl) (*, *p* < 0.0001, determined by two-way ANOVA). The horizontal bars represent the mean values ± SEM. Panel (**b**): Protein excretion showed a significant increase in WT mice treated with CDDP compared to WT control animals (NaCl) (* *p* = 0.0062, determined by unpaired Student *t*-test). However, the statistical comparison among all four groups (WT and OCT2^−/−^ mice treated with NaCl or CDDP) using a two-way ANOVA did not reveal any significant difference. The horizontal bars represent the mean values ± SEM. Panel (**c**): Glucose excretion did not show significant changes in WT and OCT2^−/−^ mice due to CDDP treatment. Control OCT2^−/−^ mice (NaCl) exhibited significantly lower glucose excretion compared to control WT mice (*, *p* = 0.0097, determined by two-way ANOVA). The horizontal bars represent the median values with the interquartile ranges. Panel (**d**): The effect of CDDP treatment on serum BUN concentration is shown. CDDP treatment significantly increased serum BUN levels only in WT mice (* *p* < 0.0001, determined by two-way ANOVA) but not in OCT2^−/−^ mice. The serum BUN concentration in OCT2^−/−^ mice after CDDP treatment was significantly different from that observed in CDDP-treated WT animals (# *p* = 0.0033, determined by two-way ANOVA).

In WT mice treated with CDDP, we observed a deterioration of renal function, as indicated by increased excretion of water (Figure 4a) and protein (Figure 4b). Conversely, increased protein excretion was not observed in OCT2^−/−^ mice (Figure 4b). Interestingly, in WT mice, renal glucose excretion (Figure 4c) was not altered by CDDP treatment compared to vehicle-treated mice, suggesting that C57BL/6J mice are less sensitive to CDDP nephrotoxicity than other strains [18]. Furthermore, a statistically significant lower renal glucose excretion was measured in OCT2^−/−^ mice compared to WT mice, suggesting a still undefined role of OCT2 for renal glucose handling. Determination of blood urea nitrogen (BUN) serum levels in mice at the end of the treatment clearly showed that CDDP caused impairment of renal function only in WT but not in OCT2^−/−^ mice (Figure 4d).

**Figure 5:**
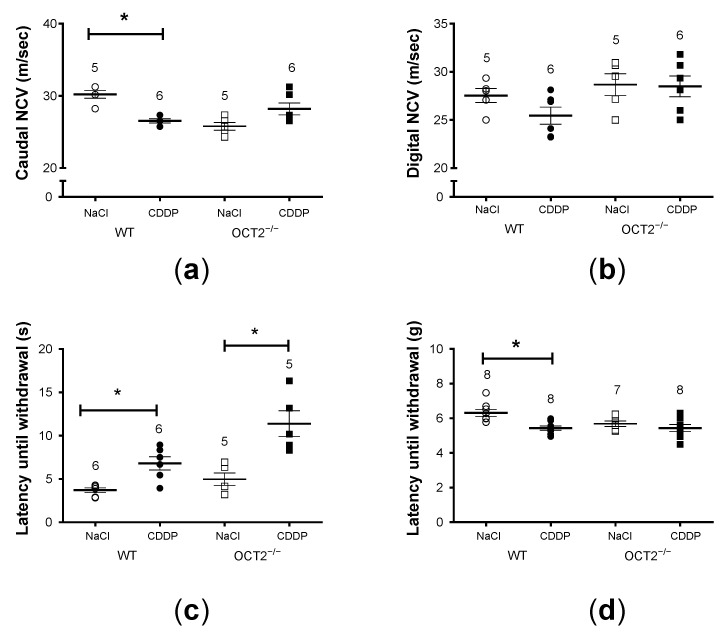
This figure shows peripheral neurotoxicity induced by CDDP treatment. A comparison is made between the effects of CDDP treatment (closed symbols) and vehicle (NaCl) treatment (open symbols) on various parameters. Panel (**a**): At the end of CDDP treatment, OCT2^−/−^ mice did not show a significant decrease in caudal nerve conduction velocity (NCV in m/s), unlike the observed decrease in C57BL/6J-WT animals (*, *p* = 0.0002, determined by two-way ANOVA). Panel (**b**): Digital NCV was not affected by CDDP treatment, as determined by two-way ANOVA. Panel (**c**): Two behavioral tests, the plantar test (panel (**c**)) and dynamic test (panel (**d**)), were conducted to assess alterations in pain perception due to chronic CDDP treatment compared to vehicle treatment. Thermal algesia was observed in both C57BL/6J-WT and OCT2^−/−^ mice (*, *p* = 0.0004, determined by two-way ANOVA, panel (**c**)). Panel (**d**): Significant mechanical allodynia was measured only in C57BL/6J-WT animals treated with CDDP (*, *p* = 0.0212, determined by two-way ANOVA, panel (**d**)). All values are presented as mean ± SEM, and the numbers above the columns indicate the number of animals measured.

Chronic CDDP treatment caused mild peripheral neurotoxicity in WT mice (Figure 5). A significant reduction of caudal nerve conduction velocity (NCV) was observed only in WT animals (Figure 5a), while digital NCV was unchanged in both WT and OCT2^−/−^ mice (Figure 5b).

**Figure 6:**
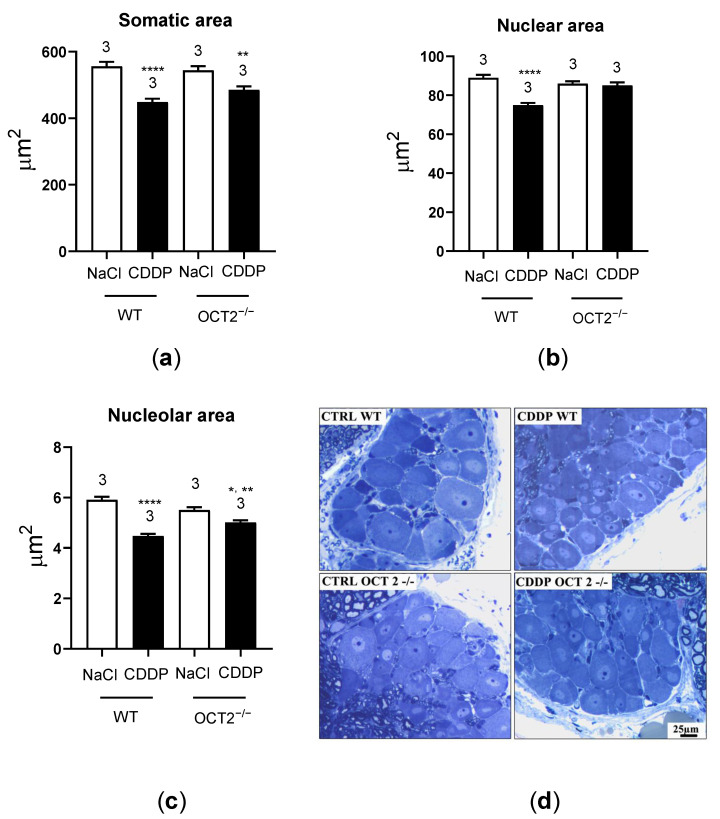
This figure shows the results of morphometric analysis of DRG (dorsal root ganglion) neurons in OCT2^−/−^ (625–627 neurons) and C57BL/6J-WT (637–647 neurons, WT) mice, with 3 animals per group. The mice were treated with either CDDP or vehicle (NaCl), and the results are presented in panels (**a**–**c**) as mean values ± SEM. Panel (**a**) shows the neuron somatic area, panel (**b**) the neuron nuclear area, and panel (**c**) the neuron nucleolar area. The findings indicate that treatment with CDDP resulted in a significant reduction in neuron somatic area in both WT (****, *p* < 0.0001, determined by two-way ANOVA, panel (**a**)) and OCT2^−/−^ animals (**, *p* = 0.003, determined by two-way ANOVA, panel (**a**)). Moreover, treatment with CDDP caused a significant reduction in neuron nuclear area exclusively in WT mice (****, *p* < 0.0001, determined by two-way ANOVA, panel (**b**)). Additionally, CDDP treatment led to a significant reduction in neuron nucleolar area in both WT (****, *p* < 0.0001, determined by two-way ANOVA, panel (**c**)) and OCT2^−/−^ animals (*, *p* = 0.01, determined by two-way ANOVA, panel (**c**)). Notably, the effect observed in WT mice was significantly greater than that observed in OCT2^−/−^ animals (**, *p* = 0.006, determined by two-way ANOVA, panel (**c**)). The numbers displayed above the columns indicate the number of animals measured in each group. (**d**) shows an example of images used for morphometric analysis.

Thermal algesia induced by CDDP treatment was observed in both WT and OCT2^−/−^ mice (Figure 5c), whereas significant mechanical allodynia was measured only in CDDP-treated C57BL/6J WT animals (Figure 5d). In addition, the dorsal root ganglia (DRG) neuron nuclear area was not significantly reduced in OCT2^−/−^ mice after CDDP treatment, while the reduction of the nucleolar area was smaller than that observed in WT animals (Figure 6). Neuron somatic area was reduced after CDDP treatment both in WT- and OCT2^−/−^ mice. Figure 6d shows an example of images used for morphometric analysis. 

**Figure 7:**
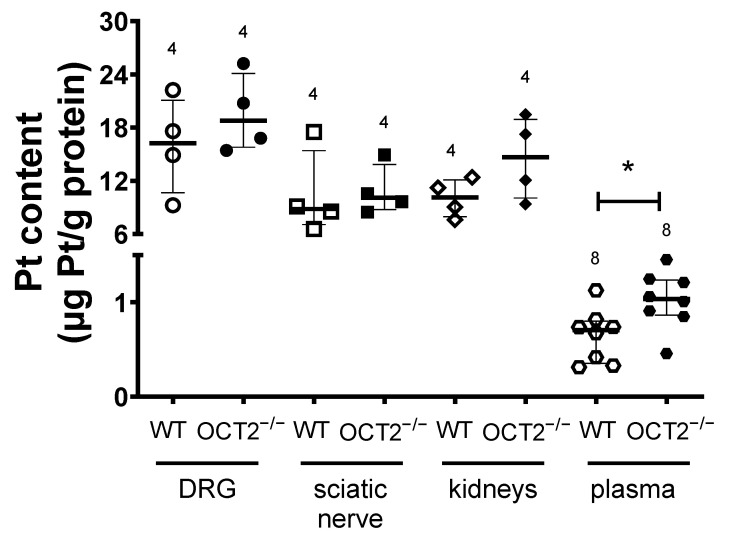
This figure shows the platinum (Pt) concentration (µg Pt/g protein) measured in various tissues, including the dorsal root ganglia (DRG), sciatic nerves, kidneys, and blood plasma, from mice undergoing chronic treatment with CDDP. The measurement was performed using flame atomic absorption spectrometry. Each symbol in the figure represents an individual animal, and the horizontal bars represent the median values with the intraquartile range. The results indicate that blood plasma samples from OCT2^−/−^ mice contain significantly higher Pt concentrations compared to samples from WT mice (* *p* = 0.02, determined by unpaired Student *t*-test). The numbers displayed above the columns indicate the number of animals measured in each group.

**Figure 8:**
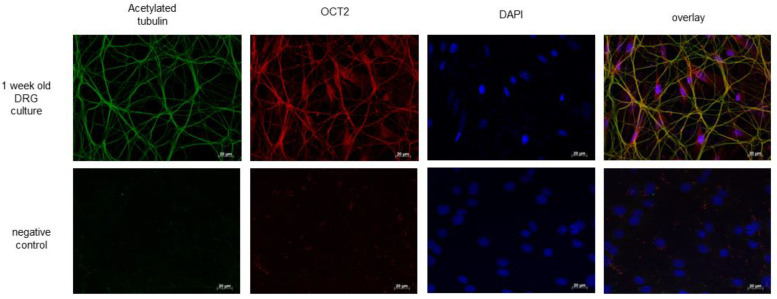
This figure shows the immunofluorescence analysis conducted to examine the expression of OCT2 in DRG (dorsal root ganglia) cultures derived from WT (wild-type) mice. The DRG cultures, which were one week old, were subjected to staining using antibodies against acetylated tubulin, serving as an axonal marker (green) and mOCT2 antibodies to visualize OCT2 expression (red). Additionally, the cell nuclei were labeled using DAPI (blue). The results revealed a clear co-localization of tubulin (green) and OCT2 (red) in the DRG cultures, as observed in the merged image (overlay). OCT2 expression was also visible in the cell bodies. In the last row of pictures, negative control experiments were conducted, where primary antibodies were omitted. These negative control images serve as a reference to confirm the specificity of the antibody staining. The scale of the images, representing the size of the structures, is indicated in the lower right corner of each picture and is 20 µm.

Quantitative measurement of platinum in DRG, sciatic nerves, and kidneys by flame atomic absorption spectrometry showed a similar concentration of platinum (Pt) in tissues from WT- and OCT2^−/−^ mice (Figure 7). Compared to WT animals, only the Pt concentration in plasma was higher in OCT2^−/−^ mice at the end of CDDP treatment (Figure 7). Immunofluorescence analysis of OCT2 expression in DRG cultures (Figure 8) shows an expression of the transporter in acetylated tubulin-positive filaments, which is probably corresponding to neurites, and cell bodies.

**Figure 9:**
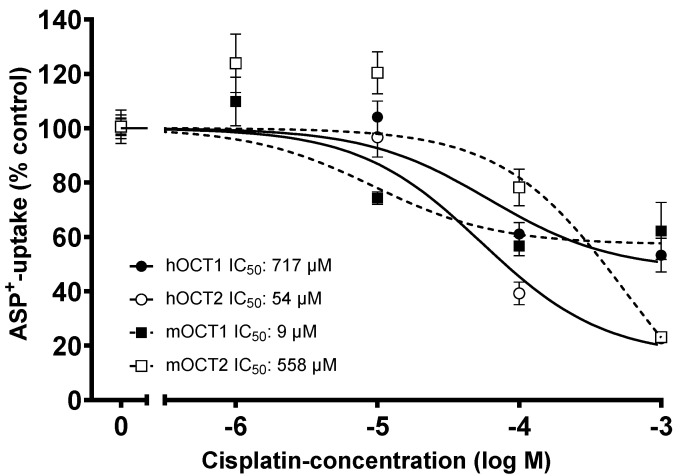
The concentration–response curves reported in this figure illustrate the inhibitory effect of CDDP on the initial uptake of ASP^+^ (a fluorescent substrate of OCT) in HEK293 cells that express different transporters: mouse OCT1 (mOCT1), mouse OCT2 (mOCT2), human OCT1 (hOCT1), or human OCT2 (hOCT2). The values presented are the mean ± SEM expressed as a percentage change of ASP^+^ uptake compared to what was measured in the absence of CDDP. The determined IC_50_ values from this curve are as follows: 9 µM for mOCT1, 558 µM for mOCT2, 717 µM for hOCT1, and 57 µM for hOCT2. The IC_50_ value represents the concentration of CDDP required to inhibit 50% of ASP^+^ uptake by the respective transporter. The logIC_50_ values, expressed as the logarithm of IC_50_, are also provided along with the corresponding standard error of the mean (SEM) and the degrees of freedom (DF) for each transporter: mOCT1: logIC_50_ = −5.0 ± 0.3 (63 DF); mOCT2: logIC_50_ = −3.3 ± 0.3 (135 DF); hOCT1: logIC_50_ = −3.1 ± 0.1 (98 DF); hOCT2: logIC_50_ = −4.2 ± 0.2 (70 DF). These values give an indication of the potency of CDDP in inhibiting ASP^+^ uptake by the respective transporters, with lower logIC_50_ values indicating higher potency.

Since the protection against CDDP nephro- and peripheral neurotoxicity was lower in OCT2^−/−^ mice than previously found in mice with genetic deletion of both OCT1 and OCT2 (OCT1-2^−/−^) [18], the interaction of CDDP with OCT1 and OCT2 function was further investigated in vitro, using the fluorescent organic cation 4-(4-dimethylaminostyryl)-N-methylpyridinium (ASP^+^) to monitor OCT function. CDDP inhibited the ASP^+^ uptake measured in human embryonic kidney (HEK) cells, stably expressing the murine (m) or human (h) OCT subtypes 1 or 2 in a concentration-dependent manner (Figure 9). Interestingly, in cells expressing the OCT1 subtypes (mOCT1 and hOCT1), maximally, 40% of ASP^+^ uptake was inhibited by 10^−3^ M CDDP, whereas in cells expressing the OCT2 subtypes, this concentration of CDDP almost completely inhibited ASP^+^ uptake. The mOCT1 displays a higher apparent affinity for CDDP than the mOCT2 (IC_50_: 9.4 µM (logIC_50_ ± SEM = −5.0 ± 0.3 with 63 degrees of freedom (DF)) and 558.3 µM (logIC_50_ ± SEM = −3.3 ± 0.3 with 135 DF) for mOCT1 and mOCT2, respectively). Conversely, hOCT1 has a lower apparent affinity for CDDP than hOCT2 (IC_50_: 717 µM (logIC_50_ ± SEM = −3.1 ± 0.1 with 98 DF) and 54.2 µM (logIC_50_ ± SEM = −4.2 ± 0.2 with 70 DF) for hOCT1 and hOCT2, respectively).

**Figure 10:**
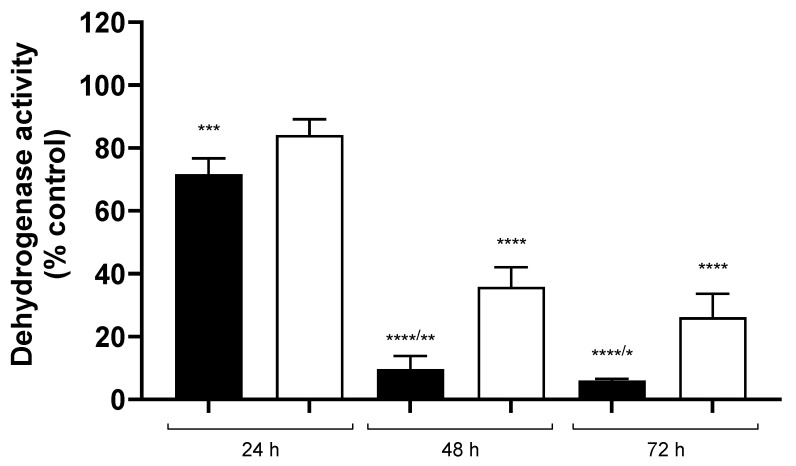
This figure shows the viability of HEK293 cells expressing mouse OCT1 (mOCT1, represented by closed columns) or mOCT2 (represented by open columns) assessed using an MTT test. The test measured dehydrogenase activity in the cells after a 10 min incubation with 100 µM CDDP, followed by post-incubation periods of 24, 48, or 72 h in normal cell culture medium. Compared to control experiments where cells were incubated for 10 min with cell culture medium without CDDP, the addition of CDDP resulted in a significant decline in cell viability. In mOCT1-expressing cells, this decline was observed already after 24 h of post-incubation time (*** *p* = 0.0002, determined by unpaired Student *t*-test). In mOCT2-expressing cells, the decline in cell viability was significant only after 48 and 72 h of post-incubation time (**** *p* < 0.0001 unpaired Student *t*-test). Furthermore, the effect observed in mOCT1-expressing cells after 48 and 72 h of post-incubation was stronger than that measured in mOCT2-expressing cells (indicated by ** and *, respectively, where ** represents *p* = 0.0026 and * represents *p* = 0.016, determined by unpaired Student *t*-test). The experiments were conducted with a total of 10 replicates in at least three independent experiments, ensuring robust data for analysis.

The higher affinity of CDDP for mOCT1 than mOCT2 was also reflected in a higher sensitivity of HEK cells stably expressing mOCT1 to cellular CDDP toxicity: significant toxicity was already evident in mOCT1-HEK cells 24 h after 10 min incubation with 100 µM CDDP (Figure 10). In mOCT2 expressing HEK cells, significant toxicity developed only 48 h after 10 min incubation with 100 µM CDDP and was less than that observed in mOCT1-HEK cells.

## 3. Discussion

Even though chemotherapy with CDDP is an efficient treatment of solid cancer, its use causes important unwanted toxicities, forcing discontinuation of CDDP administration and decreasing patients’ quality of life after CDDP-based chemotherapeutic tumor treatment. Transporters for organic cations have been demonstrated to be potential mediators of specific CDDP unwanted toxicities in both in vitro [19,20,21] and in vivo studies [15,22,23]. The in vivo investigations have been mainly performed using mice lacking both OCT1 and OCT2 (OCT1-2^−/−^) [15,18,24]. Therefore, the relative role of mouse OCT1 and OCT2 for CDDP undesired toxicities remains unclear. In this work, nephro- and peripheral neurotoxicity of cyclic repeated CDDP treatment, resembling chemotherapeutic treatment protocol of cancer patients, were studied in WT mice and in mice with genetic deletion only of OCT2 (OCT2^−/−^) to dissect the importance of this transporter for CDDP unwanted toxicities.

CDDP cyclic treatment caused a significant decrease in body weight and the weight of the testis independently from the mouse genotype, showing that these CDDP effects are not dependent on OCT2.

Nephrotoxicity at the end of CDDP treatment manifests in C57BL/6J WT mice as BUN increase and functionally as polyuria, a sign of compromised renal water resorption, and proteinuria, a sign of compromised renal filtration barrier. At the end of CDDP treatment, C57BL/6J WT mice did not develop any glucosuria, which is a sign of tubular dysfunction. Conversely, in other studies performed on the FVB mouse strain, glucosuria was an important manifestation of CDDP nephrotoxicity [18]. Compared to other strains, C57BL/6J mice seem, therefore, to be more resistant to renal insults [18,25,26].

Genetic deletion of OCT2 resulted in a milder renal phenotype, with no BUN increase and no proteinuria after CDDP treatment. However, in OCT2^−/−^ mice, CDDP-induced polyuria persists, in contrast to what was observed in [18] using OCT1-2^−/−^ animals. These results suggest that both mOCT1 and mOCT2 are involved in the development of CDDP-associated nephrotoxicity. In an attempt to translate these findings to humans, it must be considered that human kidneys mainly express hOCT2 [14,27,28].

Cyclic CDDP treatment of C57BL/6J WT mice significantly reduced caudal but not digital NCV compared to vehicle-treated mice. Indeed, other studies with chemotherapeutic agents [29] and in an acrylamide neuropathy model [30] have shown that the most distal caudal nerve is more sensitive than the digital nerve. This may be explained by the uneven cross-sectional structure and function along the axon. Cyclic CDDP treatment of OCT2^−/−^ mice did not change the caudal NCV, showing the importance of OCT2 in the peripheral neurotoxicity induced by CDDP.

The neurophysiological alterations detected through various techniques were further supported by the presence of noticeable morphological changes in the dorsal root ganglia (DRG). Morphometric analysis revealed that treatment with CDDP led to a significant decrease in the somatic area, nuclear area, and nucleolar area of the DRG. These findings provide additional evidence for the phenomenon of platinum-induced neuronal atrophy [29,31]. Changes in the organization of neuronal nuclei and alterations in neuron size have been associated with various human diseases. In particular, they have been linked to apoptotic changes observed in chronic neuropathic pain associated with nerve injury also in different rodent models [32]. These effects were absent (decrease of nuclear area) or milder (decrease of nucleolar area) in OCT2^−/−^ mice, suggesting that OCT2 deletion was able to partially protect DRG neurons from CDDP toxic effect. Using the same CDDP treatment protocol in OCT1/2^−/−^ mice [18], stronger protection from CDDP toxicity in all cellular compartments was observed compared with what was found in the present study using OCT2^−/−^ mice. Interestingly, the nucleolus was the less damaged neuronal structure by cyclic CDDP treatment in the FVB mouse strain (used in [18] as background strain), suggesting a different CDDP susceptibility of the two murine strains. Moreover, mechanical allodynia was observed only in C57Bl6/6J- (present study) and not in the FVB background (as shown in [18]). Interestingly, OCT2^−/−^ mice in a C57Bl6/6J background were protected against mechanical allodynia. Plantar test demonstrated hyperalgesia in the C57Bl6/6J WT mice following CDDP treatment. The OCT2^−/−^ mice were not significantly protected against hyperalgesia following chronic CDDP administration.

Together, these results suggest that the genetic deletion of the OCT2 transporter alone is not sufficient to completely protect the mice against CDDP-induced unwanted toxicities such as nephro- and peripheral neurotoxicity.

The expression of OCT2 in DRGs has already been demonstrated in other works [16,33]. By analyzing OCT2 expression in DRG cultures, here we have demonstrated that the transporter is present in neuron neurites. Other transporters may also be involved in determining CDDP neurotoxicity, as proposed, for example, for Ctr1 and OCTN, because of their neuronal membrane localization [34,35].

Interestingly, in contrast to previous findings after acute treatment with high concentrations of CDDP (a single bolus of 15 mg/kg body weight) [15], chronic treatment with multiple cyclic applications of 4 mg/kg body weight CDDP did not show a statistically significant difference in the accumulation of platinum (Pt) in the kidneys between WT and OCT2^−/−^ mice. This finding is intriguing as it contradicts the previous observation where renal CDDP accumulation was lower in OCT1-2^−/−^ mice compared to WT animals [15]. No statistically significant difference in Pt accumulation by WT and OCT2^−/−^ mice was also observed in peripheral nervous system specimens (i.e., sciatic nerve and DRG). However, in plasma from OCT2^−/−^ mice, a higher Pt concentration than in samples from WT animals was found, indicating that in this experimental setup of cyclic CDDP treatment, impaired renal CDDP secretion by OCT2 in OCT2^−/−^ mice causes increased Pt plasma concentration.

Comparing the apparent affinities of mouse and human OCT for CDDP, it was evident that mOCT1 has the highest affinity for CDDP. However, CDDP was not able to completely inhibit the transport function (measured as ASP^+^ uptake) of mouse and human OCT1, suggesting that OCT1 has a big binding pocket with different binding domains of different affinity for substrates, as already proposed in [36,37,38,39].

Both mOCT1 and mOCT2 can mediate CDDP cellular toxicity, even though mOCT1-mediated toxicity appears before and is stronger than that induced by mOCT2, probably because of higher mOCT1affinity for CDDP.

In conclusion, these results show that mOCT2 plays a significant role in mediating CDDP renal toxicity and peripheral neurotoxicity in an experimental setting resembling CDDP treatment of cancer patients. Direct evidence shows that OCT2 is expressed in neurites from DRG neurons. However, in mice, the mOCT1 subtype also plays an important role in these toxicities. In humans, hOCT2 is the major OCT present in renal tissue and is also significantly expressed in nervous tissue. Therefore, OCT2 may be a suitable target for protective interventions aimed at mitigating unwanted CDDP toxicities exploiting OCT2-specific inhibition.

## 4. Materials and Methods

### 4.1. Animals

The study utilized male OCT2^−/−^ mice on the C57BL/6J background obtained from Prof. Schinkel at The Netherlands Cancer Institute in Amsterdam, the Netherlands. Male C57BL/6J WT mice were obtained from the Animal Facility of the Medical Faculty in Münster. The mice used in the experiments were approximately 7 to 9 weeks old and weighed between 25 to 30 g. The colonies of mice were refreshed every 10 generations to maintain genetic integrity. It is worth noting that OCT2^−/−^ mice did not exhibit any obvious phenotypic abnormalities compared to WT mice, as stated in a previous study [40]. The experimental procedures were approved by a governmental animal welfare committee (Landesamt für Natur, Umwelt und Verbraucherschutz Nordrhein-Westfalen 84-02.04.2011.A140 and 84-02.04.2014.A454) and followed national animal welfare guidelines. The mice were housed under standard specific pathogen-free conditions with controlled environmental factors, including temperature (20–22 °C), humidity (approximately 50%), and a 12-h light/12-h dark cycle. They had free access to tap water and standard animal chow.

### 4.2. Treatment of Mice

For the treatment of mice, repeated intraperitoneal (i.p.) injections of either vehicle (normal saline, NaCl) or low doses of CDDP (4 mg/kg body weight) were administered twice a week for 4 weeks, following a therapeutic regimen similar to that used in cancer treatment for patients (Figure 1). CDDP (Teva Pharma, Ulm, Germany) was prepared freshly on the day of administration at a concentration of 1 mg/ml in isotonic saline. After the final injection of vehicle or CDDP, the mice were placed in metabolic cages to collect 24-h urine samples for measurements of glucose, protein, and water excretion. The study also evaluated the peripheral neurotoxicity of CDDP using neurophysiological and behavioral tests. After these assessments, blood samples were collected via cardiac puncture, and pressure-controlled cardiac perfusion with saline was performed through the left ventricle as described in [41]. Following perfusion, organs such as kidneys, testes, seminal vesicles, and nerves were collected and weighed. The specific number of animals used for each measurement is provided in the figures that present the results of the study.

### 4.3. Measurement of Urinary Protein and Glucose Levels and Assessment of Renal Histology

Proteins were measured using a modified Bradford method (Bradford Blue, BioRad Laboratories, Munich, Germany), and glucose was measured using the hexokinase method on a Roche Hitachi Modular automatic analyser (Gluco-quant Glucose, Roche Diagnostics, Mannheim, Germany). BUN was measured by the urease–glutamate dehydrogenase method on a Roche Diagnostic analyser (Modular P, Roche Diagnostics, Mannheim, Germany).

### 4.4. Neurotoxicity Assessments

At the end of the cyclic CDDP treatment, digital and caudal nerve conduction velocities (NCV) were measured, and behavioral tests were performed using an electromyography device (Myto2 ABN Neuro, Firenze, Italy). These tests were carried out as described in [18,42]. The behavioral tests were performed to assess the pain-like behavior of the mice as a withdrawal from a thermal and a mechanical nociceptive stimulus. The thermal nociceptive threshold of mice was measured using the plantar test (Hargreaves apparatus, No. 37370; Ugo Basile Biological Instruments, Comerio, Italy). Briefly, mice were acclimatized for 2 h in a Plexiglas chamber and then tested with a mobile infrared radiant heat source (intensity of 40IR). The heat source placed under the glass floor was positioned in the middle of the hind paw of the mouse. The time taken to move the hind paw away from the heat source was automatically measured four times (withdrawal latency) to determine the nociceptive threshold. The test lasted a maximum of 30 s to avoid thermal tissue damage and animal discomfort. Sensitivity to mechanical nociceptive stimuli after cyclic CDDP treatment was assessed using a dynamic aesthesiometer (model 37450, Ugo Basile Biological Instruments, Comerio, Italy) capable of producing a mechanical force that increases in a linear fashion. Briefly, mice were acclimatized for 2 h in a Plexiglas chamber (28 × 40 × 35 cm, wire mesh floor), and then mechanical allodynia was measured with the dynamic aesthesiometer. For this, a mechanical stimulus was applied to the plantar surface of the hind paw by means of a pointed metal filament of 0.5 mm diameter. The pressure was gradually increased to 15 g over 15 s until an apparent voluntary hind paw withdrawal response was observed. This pressure represents the mechanical nociceptive threshold index. The mechanical nociceptive threshold index was automatically recorded and expressed as the mean of three measurements per hind paw, taken alternately every 2 min. The test lasted a maximum of 30 s to avoid mechanical tissue damage and animal discomfort. Following in vivo assessments, mice were euthanized, and dorsal root ganglia (DRG) were extracted for subsequent examination of their morphology and morphometry. Specifically, the lumbar L4–L5 DRG was carefully dissected at the time of sacrifice, which occurred 48 h after the final administration of CDDP. These DRG samples were then immersed in a solution containing 2% glutaraldehyde and 4% paraformaldehyde (PFA) in 0.12 M phosphate buffer. Subsequently, the samples underwent post-fixation with OsO_4_, followed by embedding in epoxy resin. The samples were utilized for both light microscopy evaluations and morphometric analysis. For the morphometric analysis, serial 1 μm sections of the DRG were prepared, spaced 50 μm apart. Images were captured using a light microscope equipped with a camera (Leica DFC 280, Wetzlar, Germany) at a magnification of 20×. The sizes of the soma, nucleus, and nucleolus of a minimum of 625 DRG neurons per animal were manually measured and subjected to analysis using computer-assisted image analysis software (Image J version 1.52k, US National Institutes of Health, Bethesda, MD, USA). All morphometric measurements were performed by the same observer, who was unaware of the experimental conditions. To determine the total platinum (Pt) concentration, previously established methods [43] were followed using frozen samples of the sciatic nerve, DRG, kidneys, and plasma obtained from at least three animals per group at the time of sacrifice. The frozen samples were subjected to digestion with an HNO_3_-HCl solution and analyzed using flame atomic absorption spectrometry (Analyst 600 Perkin Elmer, Monza, Italy). The resulting Pt concentration, expressed in μg/g protein or μg/mL plasma, was calculated accordingly.

### 4.5. Cell Culture

For the experiments, human embryonic kidney (HEK) 293 cells that expressed either mOCT1 or mOCT2 or human OCT1-2 (hOCT1-2) in a stable manner were utilized. The process of generating and characterizing these cell lines has been detailed in previous publications [44,45]. HEK293 cells were maintained at a temperature of 37 °C in 5% CO_2_ using 50 mL cell culture flasks (Greiner, Frickenhausen, Germany). The cell culture medium consisted of Dulbecco’s minimal Eagle’s medium (Biochrom, Berlin, Germany) supplemented with 10% fetal bovine serum, 1 g/L glucose, 2 mM glutamine, 3.7 g/L NaHCO_3_, and 100 U/mL streptomycin/penicillin (Biochrom, Berlin, Germany). The selection of cells transfected with OCTs was ensured by adding 0.8 mg/mL of Geneticin (PAA Laboratories, Cölbe, Germany). The cell cultures were grown on 96-well plates until they reached 80–90% confluence. The experiments were conducted using cells from passages 20–65.

### 4.6. DRG Cell Cultures

DRG (Dorsal Root Ganglia) cell cultures were prepared following the methodology outlined in reference [46]. Initially, DRGs were carefully dissected from mice and enzymatically and mechanically dissociated. The dissociated cells were then seeded onto 1.5 cm glass coverslips and cultured in a neural growth medium for a duration of two weeks. Subsequently, the cells were transferred to culture wells with a surface area of 78 mm^2^, which had been pre-coated with poly-L-lysine, followed by collagen. The cells were maintained in an NBL medium, consisting of a neurobasal medium supplemented with B27 supplement (Invitrogen, Carlsbad, CA, USA) and 10 ng/ml nerve growth factor (NGF). For fixation, the DRG cultures were treated with 4% PFA for 30 min at room temperature (RT). Following fixation, the cultures were blocked for 2 h at RT using a blocking solution containing 10% goat serum and 0.2% Triton X-100 (Sigma-Aldrich, Taufkirchen, Germany). Primary antibodies against OCT2 (a generous gift from Prof. Koepsell [15], diluted 1:100) and class 3b-tubulin (TuJ1, diluted 1:100, Covance, Denver, PA, USA, # MMS-435P) were then applied to the cultures and incubated overnight at RT. Subsequently, the cultures were incubated with secondary antibodies, specifically goat–anti-mouse Alexa Fluor 488 (diluted 1:1000, Invitrogen, Carlsbad, CA, USA, # A28175) and goat-anti-rabbit Alexa Fluor 594 (diluted 1:1000, Invitrogen, Carlsbad, CA, USA, # A-11012). To label the nuclei, 4′,6-diamidino-2-phenylindole (DAPI, Sigma-Aldrich, Taufkirchen, Germany, # MBD0015) was used at a concentration of 1 mg/mL, diluted 1:2000 in 1% bovine serum albumin. Finally, the coverslips were inverted and mounted on glass slides using a fluorescent mounting medium (Fluoromount, Sigma-Aldrich, Taufkirchen, Germany). The DRG cultures were observed and photographed using epifluorescence microscopy (Observer Z1 with Apotome, Carl Zeiss, Oberkochen, Deutschland). Negative control slides were included, where no primary antibodies were added.

### 4.7. Fluorescence Measurements

The activity of the organic cation transporter (OCT) was assessed by measuring the uptake of the fluorescent organic cation ASP^+^ at a concentration of 1 µM, following established procedures as described in references [44,47,48]. The measurements were conducted using a microplate fluorescence reader (Infinite m200, Tecan, Crailsheim, Germany) with excitation at 465 nm and emission at 590 nm. This method allowed for the dynamic measurement of transporter activity with a high time resolution (5 s). HEK293 cells expressing different paralogs and orthologues of OCT were seeded into 96-well plates and cultured until they reached 80–100% confluence. Prior to the measurements, the cell monolayers were washed with a Ringer-like solution composed of the following (in mM): NaCl 145, K_2_HPO_4_ 1.6, KH_2_PO_4_ 0.4, D-glucose 5, MgCl_2_ 1, and calcium gluconate 1.3, with a pH adjusted to 7.4 at 37 °C. To evaluate the apparent affinities (IC_50_), which represent the concentration required to inhibit 50% of ASP^+^ uptake, the transporters’ interaction with CDDP was examined. This was achieved by measuring the cis-inhibition of ASP^+^ uptake in the presence of CDDP at concentrations ranging from 10^−6^ to 10^−3^ M, following a previously established protocol [48]. The slopes of fluorescence increase were linearly fitted and used as a measure of ASP^+^ uptake.

### 4.8. Cell Viability Test

To assess the cytotoxicity of CDDP, a modified MTT (3-(4,5-dimethylthiazol-2-yl)-2,5-diphenyltetrazolium bromide, Sigma/Merck, Darmstadt, Germany) test was employed [49]. HEK293 cells stably expressing mOCT1 or mOCT2 were cultured until they reached confluency. These cells were then incubated at 37 °C for 10 min with a Ringer-like solution containing 100 µM CDDP obtained from Teva Pharm, Ulm, Germany. The chosen concentration of 100 µM was selected as it effectively interacts with the functions of both mOCT1 and mOCT2, as indicated in Figure 9. Following the incubation, the solution was removed, and the cells were further cultured in a fresh medium for 24, 48, or 72 h. After the specified incubation period, the cells were treated with a 10 µL solution of MTT at a concentration of 5 mg/mL and incubated for three hours at 37 °C. Subsequently, the MTT solution was removed, and the cells were lysed using a solution containing 10% (*w/v*) sodium dodecyl sulfate and 40% (*v/v*) dimethylformamide. The lysates were then transferred to a new microtiter plate and allowed to incubate for an additional 90 min. The absorbance of the samples was measured at 570 nm using a multiplate reader (Infinite m200, Tecan, Crailsheim, Germany).

### 4.9. Statistics

Data were analyzed with the help of using GraphPad Prism, Version 5.0 (GraphPad Software, Inc., San Diego, CA, USA). Data are plotted as individual data points with the mean ± SEM in the case they are normally distributed, or with the median of observations and the interquartile range in the case, they are not normally distributed. Unpaired two-sided Student’s *t*-test and two-way ANOVA were used as appropriate to prove the statistical significance of the effects. Significance was inferred at the *p* < 0.05 level.

### 4.10. Chemicals

All standard chemicals have been purchased from Sigma-Aldrich (Sigma-Aldrich, Taufkirchen, Germany) at the highest purity available unless otherwise stated.

## Data Availability

Not applicable.

## References

[1] Dasari S., Njiki S., Mbemi A., Yedjou C.G., Tchounwou P.B. (2022). Pharmacological Effects of Cisplatin Combination with Natural Products in Cancer Chemotherapy. Int. J. Mol. Sci..

[2] Oun R., Moussa Y.E., Wheate N.J. (2018). The Side Effects of Platinum-Based Chemotherapy Drugs: A Review for Chemists. Dalton Trans..

[3] McMahon K.R., Rassekh S.R., Schultz K.R., Blydt-Hansen T., Cuvelier G.D.E., Mammen C., Pinsk M., Carleton B.C., Tsuyuki R.T., Ross C.J.D. (2020). Epidemiologic Characteristics of Acute Kidney Injury During Cisplatin Infusions in Children Treated for Cancer. JAMA Netw. Open.

[4] Latcha S., Jaimes E.A., Patil S., Glezerman I.G., Mehta S., Flombaum C.D. (2016). Long–Term Renal Outcomes after Cisplatin Treatment. Clin. J. Am. Soc. Nephrol..

[5] Rybak L., Mukherjea D., Ramkumar V. (2019). Mechanisms of Cisplatin-Induced Ototoxicity and Prevention. Semin. Hear..

[6] Staff N.P., Cavaletti G., Islam B., Lustberg M., Psimaras D., Tamburin S. (2019). Platinum-induced Peripheral Neurotoxicity: From Pathogenesis to Treatment. J. Peripher. Nerv. Syst..

[7] McDonald E.S., Randon K.R., Knight A., Windebank A.J. (2005). Cisplatin Preferentially Binds to DNA in Dorsal Root Ganglion Neurons in Vitro and in Vivo: A Potential Mechanism for Neurotoxicity. Neurobiol. Dis..

[8] Einhorn L.H. (2002). Curing Metastatic Testicular Cancer. Proc. Natl. Acad. Sci. USA.

[9] Marullo R., Werner E., Degtyareva N., Moore B., Altavilla G., Ramalingam S.S., Doetsch P.W. (2013). Cisplatin Induces a Mitochondrial-ROS Response That Contributes to Cytotoxicity Depending on Mitochondrial Redox Status and Bioenergetic Functions. PLoS ONE.

[10] Johnson B.W., Murray V., Temple M.D. (2016). Characterisation of the DNA Sequence Specificity, Cellular Toxicity and Cross-Linking Properties of Novel Bispyridine-Based Dinuclear Platinum Complexes. BMC Cancer.

[11] Miyazawa M., Bogdan A.R., Tsuji Y. (2019). Perturbation of Iron Metabolism by Cisplatin through Inhibition of Iron Regulatory Protein 2. Cell Chem. Biol..

[12] England C.G., Miller M.C., Kuttan A., Trent J.O., Frieboes H.B. (2015). Release Kinetics of Paclitaxel and Cisplatin from Two and Three Layered Gold Nanoparticles. Eur. J. Pharm. Biopharm..

[13] Harrach S., Ciarimboli G. (2015). Role of Transporters in the Distribution of Platinum-Based Drugs. Front. Pharmacol..

[14] Motohashi H., Nakao Y., Masuda S., Katsura T., Kamba T., Ogawa O., Inui K.-I. (2013). Precise Comparison of Protein Localization among OCT, OAT, and MATE in Human Kidney. J. Pharm. Sci..

[15] Ciarimboli G., Deuster D., Knief A., Sperling M., Holtkamp M., Edemir B., Pavenstädt H., Lanvers-Kaminsky C., am Zehnhoff-Dinnesen A., Schinkel A.H. (2010). Organic Cation Transporter 2 Mediates Cisplatin-Induced Oto- and Nephrotoxicity and Is a Target for Protective Interventions. Am. J. Pathol..

[16] Sprowl J.A., Ciarimboli G., Lancaster C.S., Giovinazzo H., Gibson A.A., Du G., Janke L.J., Cavaletti G., Shields A.F., Sparreboom A. (2013). Oxaliplatin-Induced Neurotoxicity Is Dependent on the Organic Cation Transporter OCT2. Proc. Natl. Acad. Sci. USA.

[17] Perše M. (2021). Cisplatin Mouse Models: Treatment, Toxicity and Translatability. Biomedicines.

[18] Hucke A., Rinschen M.M., Bauer O.B., Sperling M., Karst U., Köppen C., Sommer K., Schröter R., Ceresa C., Chiorazzi A. (2019). An Integrative Approach to Cisplatin Chronic Toxicities in Mice Reveals Importance of Organic Cation-Transporter-Dependent Protein Networks for Renoprotection. Arch. Toxicol..

[19] Ciarimboli G., Ludwig T., Lang D., Pavenstädt H., Koepsell H., Piechota H.-J., Haier J., Jaehde U., Zisowsky J., Schlatter E. (2005). Cisplatin Nephrotoxicity Is Critically Mediated via the Human Organic Cation Transporter 2. Am. J. Pathol..

[20] Filipski K.K., Mathijssen R.H., Mikkelsen T.S., Schinkel A.H., Sparreboom A. (2009). Contribution of Organic Cation Transporter 2 (OCT2) to Cisplatin-Induced Nephrotoxicity. Clin. Pharmacol. Ther..

[21] Yonezawa A., Masuda S., Yokoo S., Katsura T., Inui K.-I. (2006). Cisplatin and Oxaliplatin, but Not Carboplatin and Nedaplatin, Are Substrates for Human Organic Cation Transporters (SLC22A1-3 and Multidrug and Toxin Extrusion Family). J. Pharmacol. Exp. Ther..

[22] Yonezawa A., Masuda S., Nishihara K., Yano I., Katsura T., Inui K. (2005). Association between Tubular Toxicity of Cisplatin and Expression of Organic Cation Transporter ROCT2 (Slc22a2) in the Rat. Biochem. Pharmacol..

[23] Pabla N., Gibson A.A., Buege M., Ong S.S., Li L., Hu S., Du G., Sprowl J.A., Vasilyeva A., Janke L.J. (2015). Mitigation of Acute Kidney Injury by Cell-Cycle Inhibitors That Suppress Both CDK4/6 and OCT2 Functions. Proc. Natl. Acad. Sci. USA.

[24] Sprowl J.A., Lancaster C.S., Pabla N., Hermann E., Kosloske A.M., Gibson A.A., Li L., Zeeh D., Schlatter E., Janke L.J. (2014). Cisplatin-Induced Renal Injury Is Independently Mediated by OCT2 and P53. Clin. Cancer Res..

[25] Sears S.M., Sharp C.N., Krueger A., Oropilla G.B., Saforo D., Doll M.A., Megyesi J., Beverly L.J., Siskind L.J. (2020). C57BL/6 Mice Require a Higher Dose of Cisplatin to Induce Renal Fibrosis and CCL2 Correlates with Cisplatin-Induced Kidney Injury. Am. J. Physiol.-Ren. Physiol..

[26] Rabe M., Schaefer F. (2016). Non-Transgenic Mouse Models of Kidney Disease. Nephron.

[27] Motohashi H., Sakurai Y., Saito H., Masuda S., Urakami Y., Goto M., Fukatsu A., Ogawa O., Inui K.-I. (2002). Gene Expression Levels and Immunolocalization of Organic Ion Transporters in the Human Kidney. J. Am. Soc. Nephrol..

[28] Oswald S., Müller J., Neugebauer U., Schröter R., Herrmann E., Pavenstädt H., Ciarimboli G. (2019). Protein Abundance of Clinically Relevant Drug Transporters in The Human Kidneys. Int. J. Mol. Sci..

[29] Carozzi V.A., Canta A., Oggioni N., Sala B., Chiorazzi A., Meregalli C., Bossi M., Marmiroli P., Cavaletti G. (2010). Neurophysiological and Neuropathological Characterization of New Murine Models of Chemotherapy-Induced Chronic Peripheral Neuropathies. Exp. Neurol..

[30] Schaumburg H.H., Zotova E., Raine C.S., Tar M., Arezzo J. (2010). The Rat Caudal Nerves: A Model for Experimental Neuropathies. J. Peripher. Nerv. Syst..

[31] Cavaletti G., Tredici G., Marmiroli P., Petruccioli M.G., Barajon I., Fabbrica D. (1992). Morphometric Study of the Sensory Neuron and Peripheral Nerve Changes Induced by Chronic Cisplatin (DDP) Administration in Rats. Acta Neuropathol..

[32] Lessans S., Lassiter C.B., Carozzi V., Heindel P., Semperboni S., Oggioni N., Chiorazzi A., Thompson C., Wagner M., Holden J. (2019). Global Transcriptomic Profile of Dorsal Root Ganglion and Physiological Correlates of Cisplatin-Induced Peripheral Neuropathy. Nurs. Res..

[33] Huang K.M., Leblanc A.F., Uddin M.E., Kim J.Y., Chen M., Eisenmann E.D., Gibson A.A., Li Y., Hong K.W., DiGiacomo D. (2020). Neuronal Uptake Transporters Contribute to Oxaliplatin Neurotoxicity in Mice. J. Clin. Investig..

[34] Jong N.N., Nakanishi T., Liu J.J., Tamai I., McKeage M.J. (2011). Oxaliplatin Transport Mediated by Organic Cation/Carnitine Transporters OCTN1 and OCTN2 in Overexpressing Human Embryonic Kidney 293 Cells and Rat Dorsal Root Ganglion Neurons. J. Pharmacol. Exp. Ther..

[35] Liu J.J., Jamieson S.M.F., Subramaniam J., Ip V., Jong N.N., Mercer J.F.B., McKeage M.J. (2009). Neuronal Expression of Copper Transporter 1 in Rat Dorsal Root Ganglia: Association with Platinum Neurotoxicity. Cancer Chemother. Pharmacol..

[36] Koepsell H. (2019). Multiple Binding Sites in Organic Cation Transporters Require Sophisticated Procedures to Identify Interactions of Novel Drugs. Biol. Chem..

[37] Keller T., Gorboulev V., Mueller T.D., Dötsch V., Bernhard F., Koepsell H. (2019). Rat Organic Cation Transporter 1 Contains Three Binding Sites for Substrate 1-Methyl-4-phenylpyridinium per Monomer. Mol. Pharmacol..

[38] Popp C., Gorboulev V., Müller T.D., Gorbunov D., Shatskaya N., Koepsell H. (2005). Amino Acids Critical for Substrate Affinity of Rat Organic Cation Transporter 1 Line the Substrate Binding Region in a Model Derived from the Tertiary Structure of Lactose Permease. Mol. Pharmacol..

[39] Ciarimboli G., Koepsell H., Iordanova M., Gorboulev V., Dürner B., Lang D., Edemir B., Schröter R., Van Le T., Schlatter E. (2005). Individual PKC-Phosphorylation Sites in Organic Cation Transporter 1 Determine Substrate Selectivity and Transport Regulation. J. Am. Soc. Nephrol..

[40] Jonker J.W., Wagenaar E., Van Eijl S., Schinkel A.H. (2003). Deficiency in the Organic Cation Transporters 1 and 2 (Oct1/Oct2 [Slc22a1/Slc22a2]) in Mice Abolishes Renal Secretion of Organic Cations. Mol. Cell. Biol..

[41] Gage G.J., Kipke D.R., Shain W. (2012). Whole Animal Perfusion Fixation for Rodents. J. Vis. Exp..

[42] Marmiroli P., Riva B., Pozzi E., Ballarini E., Lim D., Chiorazzi A., Meregalli C., Distasi C., Renn C.L., Semperboni S. (2017). Susceptibility of Different Mouse Strains to Oxaliplatin Peripheral Neurotoxicity: Phenotypic and Genotypic Insights. PLoS ONE.

[43] Canta A., Chiorazzi A., Carozzi V., Meregalli C., Oggioni N., Sala B., Crippa L., Avezza F., Forestieri D., Rotella G. (2011). In Vivo Comparative Study of the Cytotoxicity of a Liposomal Formulation of Cisplatin (Lipoplatin^TM^). Cancer Chemother. Pharmacol..

[44] Massmann V., Edemir B., Schlatter E., Al-Monajjed R., Harrach S., Klassen P., Holle S.K., Sindic A., Dobrivojevic M., Pavenstädt H. (2014). The Organic Cation Transporter 3 (OCT3) as Molecular Target of Psychotropic Drugs: Transport Characteristics and Acute Regulation of Cloned Murine OCT3. Pflugers Arch..

[45] Harrach S., Haag J., Steinbüchel M., Schröter R., Neugebauer U., Bertrand J., Ciarimboli G. (2022). Interaction of Masitinib with Organic Cation Transporters. Int. J. Mol. Sci..

[46] Röhr D., Halfter H., Schulz J.B., Young P., Gess B. (2017). Sodium-Dependent Vitamin C Transporter 2 Deficiency Impairs Myelination and Remyelination after Injury: Roles of Collagen and Demethylation. Glia.

[47] Ciarimboli G., Schlatter E. Organic Cation Transport Measurements Using Fluorescence Techniques, Bönisch H. (2016). , Sitte, H.H., Eds..

[48] Wilde S., Schlatter E., Koepsell H., Edemir B., Reuter S., Pavenstädt H., Neugebauer U., Schröter R., Brast S., Ciarimboli G. (2009). Calmodulin-Associated Post-Translational Regulation of Rat Organic Cation Transporter 2 in the Kidney Is Gender Dependent. Cell Mol. Life Sci..

[49] Mosmann T. (1983). Rapid Colorimetric Assay for Cellular Growth and Survival: Application to Proliferation and Cytotoxicity Assays. J. Immunol. Methods.

